# Diagnostic inflation in autism spectrum disorder: an epistemological and methodological reappraisal

**DOI:** 10.3389/fpsyt.2026.1847929

**Published:** 2026-07-06

**Authors:** Luigi Croce, Irene Fusaro

**Affiliations:** 1 CeDisMa — Centre for Research on Disability and Marginality, Università Cattolica del Sacro Cuore, Milan, Italy; 2 Fondazione A18 per l’Autismo, Cagliari, Italy

**Keywords:** assessment methodology, autism spectrum disorder, diagnostic validity, differential diagnosis, female phenotype, personality disorders, sensory processing, Type I/Type II autism

## Abstract

**Background:**

The reported prevalence of Autism Spectrum Disorder (ASD) has risen nearly fourfold over two decades, fuelling debate about whether DSM-5 boundaries still demarcate a coherent clinical category. Between 2020 and 2025, multiple independent research groups have produced a convergent body of critical reflection on this question.

**Aim:**

This narrative review is an argumentative reappraisal addressing three convergent failures — classificatory, sociocultural, and methodological — and asking what minimum evidentiary standards should govern adult ASD differential diagnosis in complex cases. Four sub-themes traditionally treated separately (sensory profile, female phenotype, personality-disorder differential diagnosis, care-pathway implications) are presented as convergent illustrations of one underlying problem.

**Methods:**

Narrative integration of peer-reviewed publications (2015–2026) on ASD diagnostic validity, phenotypic and genetic heterogeneity (Type I/Type II partition; Litman et al. SPARK analysis), sensory processing specificity, female phenotype and camouflaging, and differential diagnosis with seven conditions: Borderline, Avoidant, and Schizotypal Personality Disorders; Complex PTSD; ADHD with affective dysregulation; Bipolar Spectrum; OCD-spectrum disorders; and adult disorganised attachment. Literature-identification methods and AI-assisted search with author verification are detailed in Section 1.3.

**Findings:**

The category aggregates at least two neurobiologically distinct phenotypes — Type I, prototypical, often syndromic, with high genetic load; and Type II, polygenically driven, milder, overlapping with general psychopathology — differentially affected by routine-assessment limitations. The DSM-5 sensory criterion, though neurobiologically grounded, lacks diagnostic specificity. The cross-sectional, single-source, self-report-based model dominating practice is structurally inadequate for Type II presentations and the female phenotype.

**Recommendations:**

Six minimum standards are specified: structured developmental history with ≥2 informants; multi-context behavioural observation; neuropsychological profiling; granular sensory assessment by modality, direction, and contextual stability; systematic evaluation of alternative diagnoses; and longitudinal formulation with revisability. Specialised pathways should use stepped multidisciplinary triage when differential diagnosis remains unresolved, directing individuals to appropriate parallel or alternative services rather than denying care. A minimum feasible standard for under-resourced settings is articulated alongside the ideal one.

**Conclusion:**

Restoring diagnostic specificity to ASD is not opposed to the neurodiversity framework. It is the precondition for ensuring that the diagnostic label, when applied, identifies a population for which evidence-based interventions exist and the care pathway is appropriate.

## 1 Introduction

The clinical category of Autism Spectrum Disorder (ASD) has undergone a remarkable transformation over the past three decades. Its reported prevalence has increased from approximately 1 in 150 children in the year 2000 to 1 in 36 children in the United States in 2023 (1), a trajectory without precedent in psychiatric epidemiology. This paper does not argue that ASD is a diagnostic artefact, nor that the suffering of individuals who receive the diagnosis is fabricated. It argues, rather, that the current category — as defined by DSM-5 (2) and operationalised by routine clinical practice — has become semantically unstable in ways that produce clinically consequential errors: misdiagnosis in both directions, deprivation of evidence-based treatments, and the crystallisation of incorrect diagnostic identities that resist revision.

Several factors may contribute to contemporary diagnostic uncertainty in ASD, including broader DSM-5 boundaries, increased public visibility of autism, growing use of self-report tools in adult services, and the clinical challenge of differentiating ASD from other psychiatric presentations with overlapping social, affective, and sensory features. The central problem is therefore not diagnostic expansion per se, but the mismatch between diagnostic complexity and the methodological thinness of many routine adult assessments. This review focuses on that mismatch.

### 1.1 Aim of the review and positioning relative to the existing literature

This narrative review is not a comprehensive survey of the ASD literature, of which several authoritative examples have appeared in the last five years (3–5). Those reviews systematise the state of the evidence on aetiology, diagnosis, comorbidity, and intervention; the present work has a deliberately narrower and complementary scope. It addresses a specific epistemological problem — the progressive loss of categorical specificity of the ASD construct — and operationalises it as a single core question: what minimum evidentiary standards should govern adult ASD differential diagnosis in complex cases?

(i) The review is argumentative rather than descriptive, taking a position on the diagnostic-drift debate and engaging counter-arguments explicitly (Section 11.1). (ii) It integrates the recent person-centred genetic evidence (6) and the Type I/Type II neurobiological partition (Section 5b) as anchors for the argument that ASD is internally heterogeneous in a clinically relevant way. (iii) It derives explicit minimum-quality standards for adult assessment, articulated in two complementary forms — an ideal six-component protocol and a minimum feasible standard intended for resource-constrained settings (Sections 10.7, 10.8; **Table 1**).

**Table 1:** Two-tier minimum evidentiary standards for adult ASD differential diagnosis in complex cases.

Component	Ideal standard (§ 10.7)	Minimum feasible standard (§ 10.8)
Developmental history	Structured collection across multiple domains and phases from at least two independent informants; documentary materials (school reports, family videos, prior assessments) where available; ADI-R use recommended	At least one independent informant; explicit documentation of absence or inaccessibility of further informants; non-instrumented but systematic developmental review
Behavioural observation	Multi-context observation: ADOS-2 in standardised setting plus naturalistic observation across at least two further contexts (clinical, family, occupational/educational)	Single clinical observation with explicit acknowledgement of context limitation; structured behavioural notes covering pragmatic, sensory, and interactional dimensions
Neuropsychological profiling	Assessment of executive function, central coherence, mentalising, and working memory where indicated for the clinical question	Screening-level cognitive assessment with referral if profile suggests need for specialist evaluation
Sensory assessment	Granular assessment by modality, direction (hyper/hypo/seeking), and contextual stability; structured sensory history with multi-informant corroboration; psychophysiological measures where available	Structured sensory history covering modality, direction, and contextual stability; multi-informant corroboration where possible; explicit recognition of self-report limitations
Alternative diagnoses	Systematic evaluation of differential conditions identified in Section 8 with documented reasoning for retention or exclusion	Systematic consideration of at least three alternative diagnoses appropriate to the presentation (typically: one Personality Disorder, one trauma-related, one mood or attentional condition), with documented reasoning
Longitudinal formulation	Diagnosis treated as a working clinical model; explicit criteria for re-evaluation; institutional willingness to revise in light of treatment response	No first-session diagnostic closure if any of the three alternatives remains unresolved; documented follow-up appointment within a defined time window for re-evaluation
Care-pathway logic	Stepped triage, parallel referral, provisional formulation, revisability (Section 9.1); presupposes accessible alternative services	Provisional formulation and continued follow-up within the assessing service where alternative services are absent or inaccessible; never denial of care

The ideal protocol (Section 10.7) applies where institutional resources permit. The minimum feasible standard (Section 10.8) preserves the substantive principle of the ideal protocol in resource-constrained settings without specialised ASD instrumentation. Denial of care is never an appropriate response to diagnostic uncertainty under either standard.

The four sub-themes that surface most prominently in the body of the review — sensory profile, female phenotype, personality-disorder differential diagnosis, and care-pathway implications — are presented as four convergent illustrations of how the proposed standards apply across different clinical contexts and populations, not as an enumeration.

### 1.2 Why these issues matter: a dual diagnostic challenge

The contemporary diagnostic challenge in adult ASD is dual, not single. On one side stands under-diagnosis in historically missed groups — adult women, ethnic and linguistic minorities, individuals with high cognitive functioning and adaptive camouflaging — whose recognition has been one of the major clinical achievements of the past fifteen years. On the other side stands over-diagnosis or misdiagnosis in adult psychiatric presentations whose social, affective, and sensory features overlap substantially with the ASD construct but are more parsimoniously accounted for by Personality Disorders, Complex PTSD, ADHD with affective dysregulation, Bipolar Spectrum conditions, or adult sequelae of disorganised attachment. Each risk has clinical, ethical, and service-organisational consequences; neither can be addressed by tightening or loosening diagnostic practice in isolation.

**Figure 1:**
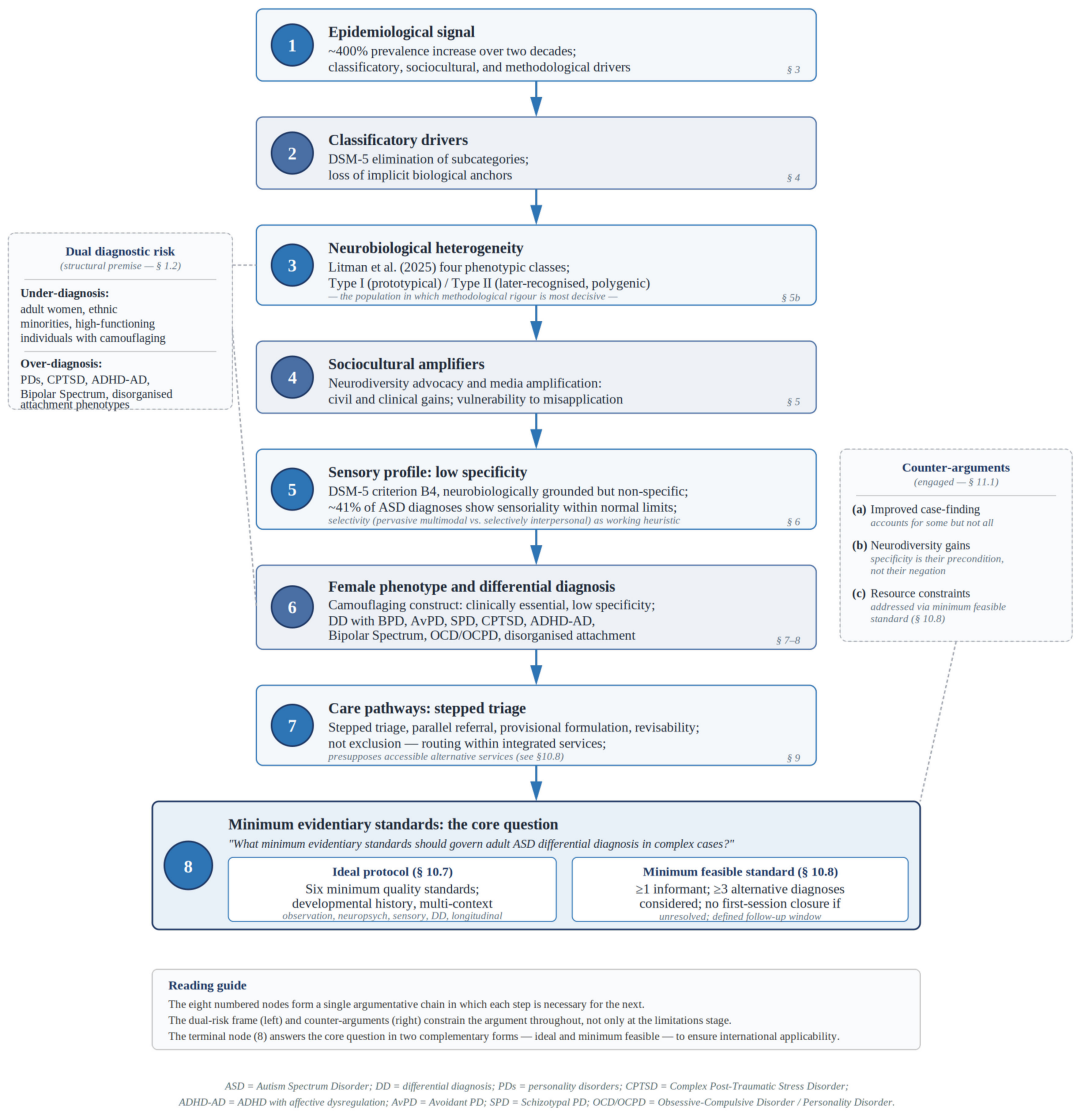
Argumentative chain of the present review, from epidemiological signal to minimum evidentiary standards in adult ASD differential diagnosis. The eight numbered nodes form a single argumentative chain in which each step is necessary for the next. The dual-risk frame (left annotation) and the counter-arguments engaged at the limitations stage (right annotation) constrain the argument throughout, not only locally. The terminal node (7) answers the core question in two complementary forms — ideal (Section 10.7) and minimum feasible (Section 10.8) — to support international applicability. ASD, Autism Spectrum Disorder; DD, differential diagnosis; PDs, personality disorders; CPTSD, Complex Post-Traumatic Stress Disorder; ADHD-AD, ADHD with affective dysregulation; AvPD, Avoidant Personality Disorder; SPD, Schizotypal Personality Disorder; OCD/OCPD, Obsessive-Compulsive Disorder/Obsessive-Compulsive Personality Disorder.

The issues addressed in this review have not been selected as a thematic catalogue but as the steps of a single argumentative chain. The argument moves from the macroscopic phenomenon (epidemiological signal) to its classificatory drivers (DSM-5 revision and absence of biological anchors), to the recent neurobiological evidence demonstrating that the category aggregates distinct phenotypes (Type I/Type II), to its sociocultural amplifiers (neurodiversity advocacy and social media), to a critical examination of one specific diagnostic criterion (the sensory profile) chosen because it exemplifies the problem of low specificity, then to the population in which low specificity has the most clinically dangerous consequences (women evaluated for ASD versus Personality Disorders), and finally to the methodological remedy (integrated assessment standards, in both ideal and minimum-feasible form). Each section is necessary for the next, and the omission of any one would leave the argument incomplete. **Figure 1** maps this argumentative chain visually and serves as a navigation aid for the rest of the manuscript.

### 1.3 Method of literature identification and selection

This is a narrative review, not a systematic one. Given its normative scope, however, the literature-identification process is made explicit here so that readers can assess whether the review is balanced or selectively assembled (**Figure 1**).

Databases searched: PubMed/MEDLINE, PsycINFO, Scopus, Web of Science, and the Cochrane Library, supplemented by hand-searching of references in the identified key papers and in the most recent narrative reviews (4, 5). Date range: 2015 to February 2026, with selective inclusion of earlier foundational works where theoretically necessary (8–10). Language restrictions: English; Italian-language sources were retained only where they report Italian service-organisation data directly relevant to the care-pathway argument. Five conceptual domains organised the search-term clusters: (a) ASD diagnostic validity and prevalence; (b) sensory processing in ASD and differential conditions; (c) female phenotype and camouflaging; (d) differential diagnosis with personality disorders, ADHD, bipolar spectrum, OCD, and trauma-related conditions; (e) service organisation and care-pathway evidence.

Inclusion principles: peer-reviewed empirical or conceptual contributions; preference for primary studies, systematic reviews, and consensus statements; editorials and commentary were retained only where they marked an explicit position in the diagnostic-drift debate and are identified as such in the text. Grey literature, advocacy materials, and non-peer-reviewed online content were not used as evidence.

The role of AI-assisted search is described in the Generative AI Statement at the end of the manuscript. In summary: AI tools were used to broaden initial coverage of the differential-diagnosis literature (Sections 8.2–8.8) and to retrieve and collate the published statements of authors active in the diagnostic-drift debate between 2020 and 2025. Each citation retrieved through this process was manually verified against the original source by the authors before inclusion; no reference was retained on the sole basis of AI suggestion. We accept that AI-assisted search raises, rather than lowers, the bar for reproducibility and source verification; this section is intended to discharge that obligation.

## 2 Convergent critical reappraisals of the ASD construct (2020–2025)

The debate on the validity of current ASD diagnostic boundaries has produced, between 2020 and 2025, a convergent body of critical reflection across multiple independent research groups. Treating this as the work of a single author would misrepresent the epistemic structure of the field. The argument of the present review draws on this collective trajectory and treats convergence across independent research groups, rather than the authority of any individual contributor, as the relevant epistemic warrant.

### 2.1 The classificatory critique

Lord and colleagues, in their Nature Reviews Disease Primers synthesis (3), signalled that the diagnostic reliability of ASD in routine clinical practice is moderate at best — well below what one would expect from a category that orients lifelong trajectories. Hirota and King (4), in their JAMA review of ASD as a clinical syndrome, documented the heterogeneity of presentations now subsumed under the unitary diagnosis. Happé and Frith, in the Annual Research Review (11), characterised the ASD construct as semantically unstable — a formulation widely cited in the subsequent literature precisely because it captures, in compact form, a worry shared across the field. Mottron (12) proposed an explicit return to diagnostic prototypes, arguing that the current dimensional drift makes biomarker discovery structurally difficult and biases the genetics of the condition towards null findings.

### 2.2 The methodological critique

Frith (13), in a brief but pointed Autism Research commentary, identified diagnosis itself as an obstacle to research: diagnostic categories based on self-report questionnaires with high sensitivity and low specificity, in the absence of direct behavioural observation and systematic developmental history, produce heterogeneous diagnostic populations that obstruct the identification of neurocognitive mechanisms specific to autism. The same critique was developed in different terms by Volkmar and McPartland (14), who reviewed the structural limitations of the ADOS-2 and ADI-R when used outside specialised research centres, and by Sonuga-Barke and colleagues (15), who raised parallel concerns about diagnostic inflation in ADHD that bear directly on the methodological argument developed here.

### 2.3 The female-phenotype and lifespan critique

Lai and colleagues (5) examined the implications of the female-phenotype literature for diagnostic boundaries, arguing that the legitimate clinical recognition of women previously missed by male-normed criteria has been accompanied by an uncritical extension of the construct to presentations with a more parsimonious alternative explanation. Bargiela and colleagues (16) and Hull and colleagues (17) developed the camouflaging construct in directions that are clinically illuminating but, as Milner and colleagues (18) have shown, lack categorical specificity for ASD. The implication, made explicit in Section 7, is that the same advance in diagnostic sensitivity has been accompanied by a loss of specificity in the same population — a phenomenon that does not call the female phenotype itself into question but raises the question of how it is operationalised in routine assessment.

### 2.4 The neurobiological critique

Litman and colleagues (6), in a person-centred analysis of the SPARK cohort (n = 5,392), identified four phenotypically distinct classes with significantly different polygenic architectures, *de novo* mutation rates, and developmental trajectories. Lombardo and colleagues (19) had earlier proposed a stratified neuroimaging framework consistent with internal heterogeneity. Robinson and colleagues (20) had documented partly distinct genetic architectures across the spectrum. The convergence of these neurobiological findings with the clinical and methodological critiques motivates the Type I/Type II partition discussed in Section 5b.

### 2.5 The advocacy-and-media dimension

The neurodiversity movement has produced civil and clinical gains that any analysis of diagnostic drift must acknowledge: reduced stigma, positive identity narratives, and the promotion of reasonable accommodation in school and workplace settings (21). Media amplification has, however, produced documented distortions: an estimated 11.5 billion views of #autism-tagged videos on TikTok in a single day, of which only one third was assessed as informationally accurate (22). The implication is not that advocacy and clinical rigour are opposed, but that a category whose social meaning has expanded faster than its scientific definition becomes structurally vulnerable to misapplication.

## 3 The epidemiological signal and its interpretation

The CDC’s 2023 surveillance data (1) document a prevalence of 2.78% in 8-year-old children in the United States — an increase of nearly 400% since the year 2000. Similar trends have been documented in Europe: Denmark, with some of the world’s most complete national registries, recorded a 400% increase in ASD prevalence between 1995 and 2010 (23). This trajectory is not explained by biological factors alone.

The literature converges on attributing the majority of the increase to non-biological factors: category expansion, stigma reduction, systemic incentives, diagnostic substitution, and improved case-finding in previously underserved populations (women, adults, ethnic and linguistic minorities). Rutter’s methodological review (24) estimated that the biologically attributable fraction of the increase is real but modest relative to the diagnostic-classificatory fraction. Hansen and colleagues’ (23) analysis of the Danish national registries attributed approximately 60% of the increase to changes in reporting practices alone.

A converging set of indirect indicators — the magnitude and rate of the reported prevalence increase, the demographic redistribution of new diagnoses towards adult and high-functioning populations, and the documented expansion of self-administered screening tools — is consistent with the hypothesis of diagnostic drift, although direct empirical demonstration remains methodologically demanding and, to our knowledge, has not yet been undertaken at the level of rigour the question requires.

As reported by Frith and Frith (22), the CDC’s own data show that 33% of diagnosed individuals have IQ below 70 and a further 24% fall in the borderline range — a distribution that is difficult to reconcile with the popular representation of autism as a condition of high sensitivity and normal cognitive profile. The mismatch between the official epidemiological profile and the social-media-mediated lay representation of the condition is itself a clinical signal worth attending to.

## 4 The DSM-5 revision: clinical gains and epistemological costs

The transition from DSM-IV-TR to DSM-5 (2) produced two taxonomically relevant modifications. The elimination of discrete subcategories — Autistic Disorder, Asperger’s Syndrome, Pervasive Developmental Disorder Not Otherwise Specified — and their convergence into the single ASD category made clinically legible populations that had previously been excluded because of atypical presentation relative to male-dominant criteria. This is a genuine clinical gain.

The simultaneous inclusion of the sensory profile as a formal criterion (Criterion B4: hyper- or hypo-reactivity to sensory input, or unusual interest in sensory aspects of the environment) has scientific grounding: neurobiological research has identified specific neurophysiological correlates consistent with weak central coherence (25) and the Bayesian predictive processing account of autism (26).

The epistemological costs, however, were underestimated. Without validated biological thresholds, the diagnosis depends entirely on clinical judgment of the degree of impairment, vulnerable to inter-individual variability, cultural bias, and contextual pressures. Lord and colleagues (3) acknowledged that the diagnostic reliability of ASD, measured with gold-standard instruments such as the ADOS-2 and ADI-R, remains moderate in real clinical contexts — substantially below what one would expect from a diagnosis that orients entire life trajectories. Volkmar and McPartland (14) reached a similar conclusion in their structural review of the gold-standard instruments, noting that inter-rater reliability degrades sharply outside specialised research centres.

## 5 The neurodiversity movement, social media, and self-diagnosis

The neurodiversity movement has produced substantive civil and clinical outcomes (21): reduced stigma, positive identity narratives, the promotion of reasonable accommodation, and — critically for the present argument — the recognition of populations that earlier diagnostic practice systematically missed, particularly adult women and individuals with high cognitive functioning. These gains are not auxiliary to the diagnostic-validity debate; they define one of the two horns of the dual challenge framed in Section 1.2.

Frith and Frith (22) documented, however, that media amplification has produced distortions of the public representation of the condition. Online narratives intercept heterogeneous experiences of suffering — trauma, personality disorders, anxiety, normative high sensitivity — offering autism as a universal explanatory framework. The sensory profile plays a particular role in this dynamic: the experience of “feeling everything more intensely” is widely presented online as a clinically informative but non-specific indicator that, in the lay register, is treated as quasi-pathognomonic of autism, when in fact it is a dimension shared by many conditions distinct from ASD.

Frith (13) identifies in this dynamic a mechanism through which diagnosis hampers research: research samples are contaminated by unverified self-diagnosed individuals whose heterogeneous profiles make it difficult to identify the biological mechanisms specific to autism and obstruct biomarker research. A socioeconomic element compounds this: in many healthcare systems, the ASD diagnosis grants access to resources — specialised educational support, healthcare exemptions, rehabilitation pathways, workplace accommodations — that would not otherwise be available, creating a structural incentive for diagnosis that distorts prevalence estimates independently of diagnostic intent. McCrossin (27) documented similar dynamics in adult assessment services in the United Kingdom, where waiting times for specialist evaluation have grown to years and the screening instruments deployed in primary care show high false-positive rates (27, 28).

### 5.1 Neurobiological evidence on internal heterogeneity: person-centred phenotypic classes and the Type I/Type II partition

The argument developed so far is classificatory and methodological. It is reinforced by a converging strand of recent neurobiological evidence demonstrating that the unitary ASD category aggregates conditions that are unlikely to share a common neuropathological core. Two partly convergent neurobiological partitions have been proposed in the recent literature, both relevant to the present argument.

#### 5.1.1 The Litman et al. person-centred phenotypic classes

Person-centred analysis of the SPARK cohort (n = 5,392) identified four phenotypic classes (6): Moderate challenges (the largest class), Broadly affected, Social/behavioural, and Mixed ASD with developmental delays. These classes differ significantly in polygenic architecture, *de novo* mutation rates, age at first diagnosable presentation, and developmental trajectory. The Broadly affected and Mixed-with-developmental-delays classes carry a substantially higher load of high-impact rare variants, broadly consistent with the historically prototypical autism phenotype. The Social/behavioural class is characterised by a polygenic profile partly overlapping with that of psychiatric conditions outside the autism spectrum (mood, anxiety, ADHD), and is over-represented amongst individuals diagnosed in adolescence or adulthood. The clinical implication is that the same diagnostic label is being applied to populations whose underlying biology differs in ways that are unlikely to be clinically inert.

#### 5.1.2 The Type I/Type II distinction

A complementary partition, increasingly discussed in the neurodevelopmental literature (19, 20, 29–31), separates an early-onset, neurobiologically anchored, often syndromic or genetically high-impact phenotype (Type I, sometimes called “prototypical” or “core” autism) from a later-recognised, milder, polygenically driven phenotype with substantial overlap with general psychopathology (Type II). Type I corresponds approximately to the historical Kanner-Asperger prototypes and to the Litman Broadly affected and Mixed-with-developmental-delays classes; it shows higher rates of intellectual disability, language delay, syndromic comorbidity, identifiable rare variants of large effect, and is recognised early in development with high diagnostic stability. Type II corresponds to a substantial portion of the recent diagnostic increase, particularly in adults and women without intellectual disability, and overlaps biologically with the Litman Social/behavioural class; it shows polygenic risk profiles that share variance with mood, anxiety, and ADHD, later age at recognition, lower diagnostic stability, and presentations in which camouflaging is operationally relevant.

#### 5.1.3 Caveat on the heuristic status of the partition

The Type I/Type II terminology is not formally codified in DSM-5-TR or ICD-11. The dichotomy is best read as a heuristic distillation of a body of evidence consistent in direction across genetic, neuroimaging, and clinical studies, rather than as a discrete taxonomy with sharp boundaries. The partition is dimensional rather than categorical, with intermediate cases that resist clean classification. We use the terminology because it captures, in compact form, a clinical reality that the unitary diagnostic category obscures, whilst explicitly flagging its heuristic status to avoid overstatement.

#### 5.1.4 Implications for the central argument

The Type I/Type II partition provides a neurobiological anchor for what the present review treats as a classificatory and methodological problem. If the diagnostic category contains at least two phenotypes with different genetic architectures, developmental trajectories, and partly different cognitive profiles, then four consequences follow.

(i) Treating the category as unitary is empirically unwarranted. Aggregate prevalence figures, biomarker searches, and treatment-outcome meta-analyses that pool Type I and Type II together are likely to produce diluted or inconsistent findings. (ii) Diagnostic instruments calibrated on Type I (ADOS-2 and ADI-R, in their original validation samples, were predominantly developed on early-onset, often male, often syndromic populations) under-perform in Type II, where camouflaging, near-normal cognition, and absence of early developmental anomalies make the standard observational signs unreliable. (iii) The differential diagnosis with Personality Disorders, Anxiety, and Complex PTSD becomes most consequential precisely in Type II, because that is where phenomenological overlap with general psychopathology is greatest. (iv) The methodological recommendations developed in Section 10 are, in substance, recommendations for distinguishing genuine Type II from non-ASD conditions presenting with similar surface features. The Type I/Type II distinction therefore reinforces rather than replaces the methodological argument: it identifies the clinical population in which methodological rigour is most decisive.

## 6 The sensory profile in ASD: nuclear component, limited specificity

### 6.1 Neurocognitive bases and DSM-5 inclusion

Sensory processing differences in ASD have been identified since the original descriptions of Kanner and Asperger (8). Their formal inclusion in DSM-5 has neurobiological grounding: the weak central coherence model (25) postulates that individuals with ASD process stimuli with a bias towards local detail rather than global gestalt, producing fragmented, hyper-focused sensory processing. The aberrant predictive processing account (26) proposes that in ASD, prediction error signals carry disproportionate weight relative to internal priors, producing amplified bottom-up processing that translates into a sensory world perceived as more chaotic and intense.

Williams and colleagues (32), in a systematic review of sensory processing in ASD across exteroceptive and interoceptive domains, documented that sensory differences concern virtually all sensory channels — auditory, visual, tactile, vestibular, proprioceptive, olfactory, gustatory, and interoceptive — with notable intra-categorical heterogeneity. Prevalence estimates for sensory differences in ASD range from 42% to 96% across studies (33), a range that reflects not only real heterogeneity but also the methodological limitations of measurement instruments and the absence of a shared taxonomy (34).

### 6.2 Intra-categorical heterogeneity and limited specificity

Kadlaskar and colleagues (35), in a latent profile analysis of 211 autistic children aged 2–4 using the Short Sensory Profile, identified four distinct sensory classes: Moderate/Mixed (35.5%), Severe/Mixed (8.5%), Moderate/Broad (14.6%), and Low/Mixed (41.1%). The final class — nearly 41% of the sample — displays sensory behaviours broadly within normal limits, suggesting that a substantial proportion of children with an ASD diagnosis does not present the atypical sensory profile that popular narratives universally associate with the disorder.

A central diagnostic challenge is that sensory differences, although clinically important in ASD, are not unique to ASD and therefore cannot be interpreted in isolation from developmental history, contextual patterning, co-occurring psychopathology, and functional profile. Hyper- or hypo-reactivity is found with high frequency in Anxiety Disorders, PTSD and Complex Trauma (ICD-11 CPTSD; (36)), Borderline Personality Disorder (BPD), ADHD, and the normative Highly Sensitive Person variant (37). Cummings and colleagues (38) demonstrated significant biological overlaps between anxiety and sensory over-responsivity in youth with ASD compared to those with anxiety disorders, rendering differential diagnosis even more dependent on longitudinal clinical history. The lay-register equation of generalised sensory intensity with autism corresponds to a meaningful self-understanding that does not, by itself, constitute a clinical diagnosis. **Table 2** presents the comparative sensory profile characteristics across the conditions posing differential diagnostic challenges.

**Table 2:** Sensory profile characteristics across ASD and the conditions posing differential diagnostic challenges.

Condition	Modality	Direction	Contextual pattern	Developmental onset
ASD (prototypical/Type I)	Pervasive, multimodal	Hyper- and hypo-reactivity often co-present, with sensation seeking	Stable across contexts, present in neutral states	Documented in early childhood by multiple informants
ASD (Type II, female phenotype)	Often multimodal but milder; partially compensated by camouflaging	Hyper-responsivity often predominant; sensation seeking less marked	Relatively stable; partial context-dependence due to compensation	May be retrospectively reconstructed; signs often missed in childhood
Borderline Personality Disorder	Selectively interpersonal; relational hyperaesthesia	Predominantly hyper-responsive within affective activation	State-dependent; reduces outside relational triggers	Typically emerges in adolescence/young adulthood with affective dysregulation
Complex PTSD/disorganised attachment	Trigger-bound; modality follows trauma cue	Hyper-responsivity to trauma-relevant stimuli; dissociative numbing possible	Strongly state-dependent; tied to reminders	Onset linked to traumatic exposure; chronology key
Anxiety Disorders	Often selective; arousal-amplified	Hyper-responsivity rising with anxiety state	State-dependent; reduces with anxiolytic response	Variable; often adolescence onward
ADHD with affective dysregulation	Selective, modulation-related	Sensation seeking + difficulty filtering input	Reduces with stimulant treatment	Childhood, but pattern differs from ASD
Highly Sensitive Person (normative)	Multimodal but mild; no functional impairment	Hyper-responsivity within normal range	Trait-stable; not pathological	Lifelong trait dimension

The contrasts presented here are clinically useful working distinctions supported by convergent observational data; head-to-head psychophysical studies remain limited, particularly for ASD versus BPD (see Section 6.3).

### 6.3 Sensory profile in ASD versus personality-disorder differential diagnosis

In the context of differential diagnosis with Personality Disorders in the female phenotype, the sensory profile assumes an ambiguous role. Women with BPD can describe intense experiences of relational hyperaesthesia — the sensation of being overwhelmed by others’ emotions, of perceiving interpersonal atmospheres in an acutely heightened way — that online narratives systematically reinterpret as indicators of masked ASD.

A clinically useful working distinction concerns selectivity: in BPD, sensory hyper-reactivity tends to be selectively interpersonal and relational, connected to emotional activation states, and to reduce in non-relational sensory contexts; in ASD, hyper- or hypo-sensory reactivity tends to be pervasive, multimodal, present across modalities and across contexts, and largely independent of relational charge. This contrast is best read as a clinically useful heuristic supported by convergent observational data, rather than as a finding already established at the level of robust differential evidence: head-to-head psychophysical studies comparing BPD and ASD on multimodal sensory profiling are limited, and **Table 3** should be read in that light. With this caveat, the relational-selectivity-versus-pervasive-multimodality contrast remains, in our clinical experience, one of the more useful differentiating elements accessible through a structured sensory history of adequate granularity.

**Table 3:** Differential diagnosis: ASD female phenotype versus Borderline Personality Disorder.

Dimension	ASD (female phenotype)	Borderline Personality Disorder
Trauma chronology	Trauma frequently secondary to chronic social non-fitting; longitudinal social-communication atypicality precedes affective sequelae	Early relational trauma etiologically central; affective dysregulation arises from disrupted attachment
Sensory profile	Pervasive, multimodal, stable across contexts; largely independent of relational charge	Selectively interpersonal hyperaesthesia; state-dependent; reduces in non-relational contexts
Identity stability	Stable but rigidly organised identity; difficulty with role flexibility	Identity diffusion; instability across relationships and time
Relational pattern	Difficulty initiating and reading social cues; not driven by abandonment fear	Intense, unstable relationships dominated by fear of abandonment
Affective regulation	Difficulty modulating arousal, often sensory-triggered; alexithymia common	Rapid affective oscillations triggered by relational events; reactive impulsivity
Theory of mind	Hypo-mentalising; mechanical/rule-based compensation strategies	Mentalising preserved overall but vulnerable under affective load; hyper-mentalising in conflict
Treatment response	Adapted CBT, psychoeducation, environmental accommodation; limited response to BPD-specific therapies	Strong evidence base for DBT, Schema Therapy, MBT; substantial remission rates documented (39)
Risk of misdiagnosis	If diagnosed as BPD: deprivation of supports for sensory and pragmatic difficulties	If diagnosed as ASD: deprivation of evidence-based BPD treatments; sensory accommodations may reinforce avoidance

The contrasts presented here are clinically useful working distinctions supported by convergent observational data and clinical experience; they are not yet established at the level of robust head-to-head differential evidence (see Section 6.3).

## 7 The female phenotype: clinical reality and diagnostic challenge

One of the most important research advances of the past fifteen years is the recognition of the female phenotype as a systematically different clinical presentation from the male phenotype, historically under-recognised (40). The clinical and ethical significance of this advance is difficult to overstate: cohorts of adult women who had spent decades in psychiatric or psychological services without an accurate diagnostic formulation have, in many cases, found in the female-phenotype literature a framework that finally accounted for their developmental trajectory. The neurodiversity framework has contributed materially to this recognition, and the present review takes that contribution as a constraint on its argument rather than as an object of critique.

Lai and colleagues (41, 42) documented camouflaging strategies — deliberate imitation of social norms, suppression of repetitive behaviours, use of learned conversational scripts — and their cost in terms of autistic burnout and comorbidities. Hull and colleagues (17) developed the Camouflaging Autistic Traits Questionnaire (CAT-Q) as an objectification instrument. Bargiela and colleagues (16) documented the human cost: chronic exhaustion, elevated anxiety, a pervasive sense of identity falsification.

The clinical problem is not the female phenotype itself, but the uncritical application of the camouflaging construct to presentations with a more parsimonious alternative explanation. Camouflaging has, in some clinical and online registers, become near-irrefutable: a woman with good adaptive capacities but significant internal pain can be read as a masking autistic almost by definition. In this framework, the sensory profile assumes a confirmatory role, with self-reported sensory sensitivity used as evidence of masking and producing a double layer of irrefutability.

Milner and colleagues (18) showed that individuals with high autistic traits but without formal diagnosis show camouflaging profiles overlapping those of diagnosed individuals — suggesting the construct measures a continuous population dimension rather than an ASD-specific marker. The CAT-Q and related camouflaging constructs may be clinically informative, but they should not be treated as disorder-specific indicators in the absence of convergent developmental and observational evidence. Lai and colleagues (5), in a more recent overview, have themselves cautioned against the uncritical use of camouflaging as a diagnostic shortcut, recommending that it be evaluated alongside developmental history and multi-context behavioural observation rather than as a stand-alone confirmatory criterion.

In the framework of the Type I/Type II partition introduced in Section 5b, the camouflaging construct is most clinically meaningful when applied to genuine Type II presentations — women with developmentally documented social-communication and sensory atypicalities who have learned to compensate. Its diagnostic value collapses when applied to women whose presentation has no developmental antecedent and whose adaptive functioning is preserved across contexts, where a personality-disorder, anxiety, or trauma-related framework provides a more parsimonious account. The dual-risk framing introduced in Section 1.2 applies here in its most concrete form: any tightening of criteria around camouflaging must be paired with structural safeguards against re-marginalising the women whose recognition the construct made possible.

## 8 Differential diagnosis between ASD and overlapping conditions

### 8.1 The differential-diagnostic landscape: scope and selection

This section addresses differential diagnosis between ASD and the conditions that, in our reading of the literature and routine clinical practice, most frequently produce diagnostic confusion in adult assessment. The list is not exhaustive: schizophrenia-spectrum disorders, intellectual disability without ASD, language disorders, eating disorders, and substance-use disorders can also enter into differential consideration in specific clinical configurations. The selection here reflects three criteria: (i) phenomenological overlap with ASD severe enough to produce error in routine assessment; (ii) divergent therapeutic implications, so that misdiagnosis has measurable clinical cost; and (iii) clinical prevalence high enough to justify systematic discussion. The conditions covered are: BPD (the most clinically consequential differential, treated in detail in Section 8.2); Avoidant Personality Disorder (AvPD) and Complex PTSD (Section 8.3); Schizotypal Personality Disorder (SPD; Section 8.4); ADHD with affective dysregulation (Section 8.5); Bipolar Spectrum Disorders (Section 8.6); Obsessive-Compulsive and Obsessive-Compulsive Personality Disorders (Section 8.7); and adult presentations of disorganised attachment/Reactive Attachment phenotypes (Section 8.8).

### 8.2 Borderline Personality Disorder: the most consequential differential

BPD represents the most complex differential-diagnostic case and the one with the most therapeutically relevant consequences in case of error. The phenomenological overlaps are real: emotional dysregulation, relational difficulties, trauma history, self-harming behaviours, social alienation. However, the underlying reasons are profoundly different, with divergent therapeutic implications. The most critical diagnostic point concerns the function and chronology of trauma: in BPD, early relational trauma is an etiologically central component of the model (39); in ASD, trauma is frequently secondary, produced by the chronic experience of social non-fitting. On the sensory level, as discussed in Section 6.3, hyper-reactivity in BPD is more selectively interpersonal whereas in ASD it tends to be pervasive and multimodal — a contrast that is clinically useful as a working distinction, with the caveats noted there.

The therapeutic consequences of misdiagnosis are severe. A woman with BPD who receives an ASD diagnosis is deprived of evidence-based treatment frameworks — Dialectical Behaviour Therapy (43), Schema Therapy (44), Mentalisation-Based Treatment (45) — that have robust outcome data in BPD. Dysfunctional behaviour is reinterpreted as neurologically determined and therefore non-modifiable, reducing both the clinician’s and the patient’s expectations of change in a self-confirming cycle. Zanarini and colleagues (39), in a 16-year longitudinal follow-up, documented that a substantial proportion of individuals with BPD achieves sustained symptomatic remission with appropriate treatment. Incorrect ASD labelling may foreclose this pathway. In addition, if sensory accommodations are proposed on the basis of a misdiagnosed sensory profile, they may reinforce avoidance patterns that are therapeutically contraindicated in BPD. **Table 3** presents the key clinical dimensions for differential diagnosis between ASD female phenotype and BPD, with the heuristic-status caveat noted in Section 6.3.

### 8.3 Avoidant Personality Disorder and Complex PTSD

AvPD shares with ASD social avoidance and rejection history. The fundamental distinction lies in motivation: in AvPD, the person intensely desires relationships but fears them because of fear of judgment, with full awareness of implicit social rules. At the sensory level, the AvPD profile is not associated with stable, multimodal hyper/hypo-sensory reactivity. Complex PTSD (ICD-11 CPTSD; (36)) produces emotional regulation difficulties, identity disturbances, and sensory hyperreactivity to triggers that may be mistaken for the ASD sensory profile; the narrative reconstruction of trauma and its temporal relationship with sensory symptoms is the discriminating element.

### 8.4 Schizotypal Personality Disorder

SPD shares with ASD social withdrawal, restricted affect, atypical communication, and unusual interests, and the differential is one of the most genuinely difficult in adult psychiatry. The relevant distinguishing features, based on the literature and routine clinical experience, are the following.

#### 8.4.1 Cognitive and perceptual content

SPD is characterised by magical thinking, ideas of reference, paranoid ideation, and unusual perceptual experiences that are phenomenologically schizophrenia-spectrum and often respond, at least partially, to low-dose antipsychotic treatment (46). ASD restricted interests, even when intense and idiosyncratic, do not have this perceptual-cognitive quality.

#### 8.4.2 Developmental trajectory

SPD typically becomes clinically evident in late adolescence or early adulthood and shows familial co-aggregation with schizophrenia-spectrum conditions. ASD social-communication difficulties are present from early childhood and have a different familial aggregation pattern.

#### 8.4.3 Sensory profile

SPD does not typically show the stable, multimodal, neutral-context sensory hyper/hypo-reactivity characteristic of ASD. Sensory atypicalities in SPD, when present, are embedded in the broader perceptual-anomaly phenomenology rather than constituting a stable trait.

#### 8.4.4 Theory of mind

Both conditions show theory-of-mind impairments, but the qualitative profile differs: SPD shows hyper-mentalising errors with paranoid colouring; ASD shows hypo-mentalising with mechanical or rule-based compensation strategies (46, 47).

#### 8.4.5 Therapeutic implications

The differential is clinically consequential because the treatment frameworks diverge: low-dose antipsychotic plus structured psychotherapy in SPD; psychoeducation, environmental accommodation, and adapted CBT in ASD. A misdiagnosis in either direction has measurable clinical cost.

### 8.5 ADHD with affective dysregulation

ADHD with marked affective dysregulation — a clinical picture that overlaps with what was formerly captured by Disruptive Mood Dysregulation Disorder in childhood and by emotional-impulsivity ADHD subtypes in adulthood — produces a phenomenology that can mimic both ASD and BPD (48). The differentiating elements with respect to ASD are: (i) preserved social reciprocity and pragmatic communication in low-arousal contexts; (ii) sensory sensitivity that is more often selective and modulation-related (sensation seeking and difficulty filtering input) than pervasive and multimodal; (iii) absence of restricted interests with the intensity, specificity, and functional absorption typical of ASD; (iv) clear response to psychostimulant trials in the ADHD core symptoms, which does not occur in pure ASD. Comorbidity ADHD + ASD is, however, well documented and prevalent (around 30–50% in clinical samples; (49)) and remains a legitimate diagnostic conclusion when both sets of criteria are independently met.

### 8.6 Bipolar spectrum disorders

Subthreshold bipolar presentations — particularly Bipolar II and ultra-rapid-cycling phenotypes — can be misread as ASD when the inter-episode functioning is marked by social withdrawal, sensory sensitivity (often mood-state-dependent), and rigidity. The differentiating element is the episodic structure: bipolar phenomenology is organised in mood episodes with clear onset, offset, and duration criteria, whilst ASD traits are continuous and trait-like (50). Family history, response to mood stabilisers, and the temporal pattern of sensory and social difficulties (state-dependent versus trait-like) are the operationally relevant discriminators. Genuine ASD + Bipolar Spectrum comorbidity exists and warrants specific management.

### 8.7 Obsessive-Compulsive Disorder and Obsessive-Compulsive Personality Disorder

Obsessive-Compulsive Disorder (OCD) and Obsessive-Compulsive Personality Disorder (OCPD) share with ASD repetitive behaviours, insistence on sameness, and rigidity. The phenomenological distinction is well established: OCD compulsions are ego-dystonic, anxiety-driven, and recognised by the patient as excessive; ASD repetitive behaviours and routines are ego-syntonic, regulatory, and produce distress only when interrupted (51). OCPD rigidity concerns moral, productivity, and relational standards rather than sensory-motor or interest-related routines. The sensory profile and the social-reciprocity dimension remain the most decisive discriminators. Genuine ASD + OCD comorbidity is documented and clinically meaningful, with specific implications for the choice between exposure-based and accommodation-based interventions.

### 8.8 Adult presentations of disorganised attachment and Reactive Attachment phenotypes

Adult presentations rooted in early disorganised attachment, or in formally diagnosed Reactive Attachment Disorder of childhood, can show social-relational atypicalities, dissociative phenomena, and emotional dysregulation that may be misread as ASD, particularly in the female phenotype (52). The differentiating elements are the trauma-history coherence (relational disruption chronologically preceding the difficulties), the presence of dissociative phenomena that respond to trauma-focused treatment, and the absence of the multimodal stable sensory profile and of restricted interests in the ASD sense. The differential is most challenging in individuals who have experienced early institutionalisation or chronic caregiver disruption and who present in adulthood with social withdrawal, communicative atypicality, and sensory sensitivity. **Table 4** summarises the extended differential diagnosis across the seven conditions discussed in this section and five clinical dimensions.

**Table 4:** Extended differential diagnosis across the seven conditions discussed in Section 8 and five clinical dimensions.

Condition	Onset/trajectory	Sensory profile	Social motivation/ToM	Episodic vs trait	Therapeutic response
BPD	Adolescence/young adulthood; relational trauma central	Selectively interpersonal hyperaesthesia	Preserved social drive; ToM preserved overall, unstable under load	Trait-like with strong state modulation	Robust response to DBT, Schema, MBT
AvPD	Adolescence onward; pervasive fear of judgement	Not multimodal stable hyper/hypo-reactivity	Strong relational desire with fear of judgement; ToM intact	Trait-like	CBT for social anxiety; gradual exposure
CPTSD (ICD-11)	Linked to traumatic exposure; chronology key	Trigger-bound; trauma-relevant	Variable social drive; ToM intact but trauma-distorted	State-dependent on triggers	Trauma-focused therapy (EMDR, CPT, narrative)
SPD	Late adolescence/early adulthood; familial schizophrenia-spectrum	Not stable multimodal; perceptual anomalies	Reduced social drive; ToM impaired with hyper-mentalising paranoid colouring	Trait-like with episodic perceptual anomalies	Low-dose antipsychotic + structured psychotherapy
ADHD with affective dysregulation	Childhood; persistent attentional core	Selective, modulation-related; sensation seeking	Preserved social reciprocity in low arousal	Trait-like, with state amplification	Psychostimulant trial; CBT; coaching
Bipolar Spectrum	Variable; family history relevant	State-dependent sensory sensitivity	Preserved social drive between episodes	Strongly episodic with clear onset/offset	Mood stabilisers; cyclothymia-specific psychoeducation
OCD/OCPD	Childhood-adolescence (OCD); adulthood (OCPD)	Not the multimodal stable profile of ASD	Preserved social reciprocity	OCD: episodic exacerbations; OCPD trait	ERP (OCD); cognitive therapy (OCPD)
Disorganised attachment/RAD-adult	Early relational disruption; institutional history common	Trigger-bound; dissociation-linked	Variable; ToM disrupted by trauma	Strongly state-dependent	Trauma-focused integrative treatment

The table is intended as a clinical heuristic supporting structured assessment, not as a substitute for longitudinal clinical formulation; head-to-head empirical evidence varies in maturity across the cells.

### 8.9 Structured differential-assessment indicators

Based on the systematic comparison developed in Sections 8.2 to 8.8, fourteen indicators consistently emerge as clinically useful for the differential assessment, summarised here for practical reference:

– (i) Trauma chronology — early relational versus secondary social-non-fitting: indicative of BPD/CPTSD versus ASD.

– (ii) Sensory pattern — pervasive multimodal stable versus selectively interpersonal mood-dependent: indicative of ASD versus BPD.

– (iii) Social motivation — desire for relationships with fear of judgement versus reduced social drive: indicative of AvPD versus ASD.

– (iv) Episodic structure — mood episodes with clear onset/offset versus continuous trait-like presentation: indicative of Bipolar Spectrum versus ASD.

– (v) Cognitive-perceptual quality — magical thinking and ideas of reference: indicative of SPD versus ASD.

– (vi) Theory-of-mind profile — hyper-mentalising paranoid versus hypo-mentalising mechanical: indicative of SPD versus ASD.

– (vii) Developmental documentability of social-communication atypicalities across childhood, adolescence, and adulthood: supportive of ASD when present from multiple independent informants.

– (viii) Restricted-interest functional absorption with intensity, specificity, and longitudinal stability: supportive of ASD.

– (ix) Pragmatic communicative anomalies in low-arousal contexts (literalism, atypical prosody, conversational rigidity): supportive of ASD.

– (x) Inter-context behavioural stability versus relationship-dependent modulation: supportive of ASD versus Personality Disorders.

– (xi) Stimulant-trial response in core attentional symptoms: supportive of ADHD as primary or comorbid.

– (xii) Dissociative phenomenology with trauma-focused treatment response: supportive of CPTSD or disorganised-attachment framework versus ASD.

– (xiii) Family-history pattern — schizophrenia-spectrum, mood, neurodevelopmental: orienting variable across SPD, Bipolar Spectrum, and ASD respectively.

– (xiv) Ego-dystonic quality of repetitive behaviours, recognised by the person as excessive: indicative of OCD.

## 9 Implications for care pathways

### 9.1 Stepped triage, parallel referral, and provisional formulation

Specialised ASD services should adopt a staged assessment principle: when differential diagnosis remains unresolved, the case should move to extended multidisciplinary evaluation or parallel referral rather than to immediate diagnostic closure. The relevant distinction is not between inclusion and exclusion, but between premature labelling and clinically responsible formulation. In practice, this means maintaining access to care whilst ensuring that ASD is confirmed only when developmental, behavioural, and differential evidence converge sufficiently.

Four operational principles structure this approach. First, stepped triage: where clear convergent evidence supports the ASD diagnosis at the initial assessment, diagnostic closure is appropriate and further assessment proceeds within the ASD pathway. Where differential alternatives remain plausible, the case is moved to a second, more articulated assessment phase rather than receiving an immediate diagnosis. Second, parallel referral: where an alternative diagnosis (BPD, AvPD, SPD, CPTSD, ADHD with affective dysregulation, Bipolar Spectrum, OCD/OCPD, disorganised-attachment phenotype) appears more parsimonious than ASD or as primary in a co-occurrence configuration, the person is referred to the appropriate service in parallel, without delay and without dismissal of the ASD hypothesis. Third, provisional formulation: where neither closure nor confident redirection is yet possible, a provisional clinical formulation is documented and the diagnostic hypothesis is held open, with explicit criteria for re-evaluation. Fourth, revisability: the ASD diagnosis, once made, is not treated as irrevocable; it is treated as a working clinical model that remains open to revision in light of treatment response, longitudinal evolution, and new information from independent informants.

This approach presupposes the existence of accessible alternative services — general adult psychiatry, personality-disorder programmes, trauma services, ADHD specialist clinics, mood-disorder programmes — to which parallel referrals can be made. Where such services are absent or inaccessible, as is the case in many fragmented and under-resourced systems, the responsibility of the assessing clinician shifts from referral to provisional formulation and continued follow-up within the assessing service. It does not, under any circumstances, become denial of care. Section 10.8 develops the implications of this constraint for international applicability.

Misdiagnosis consequences extend across multiple levels. Therapeutically, the person is deprived of evidence-based treatments. Behaviourally, dysfunctional behaviour is reinterpreted as neurologically determined and non-modifiable. Identitarily, a rigid identity consolidated around the ASD diagnosis may actively resist diagnostic revision. With respect to the sensory profile specifically, proposed accommodations may reinforce avoidance patterns rather than promoting the graduated exposure that is therapeutically indicated in anxiety and BPD. A specialised ASD pathway that systematically considers alternative diagnoses before confirming ASD — and that maintains the diagnostic hypothesis as revisable in light of treatment response — is a clinically and ethically more defensible model than one that treats the diagnosis as irrevocable. Communication with individuals and families is integral to this approach: staged assessment must be presented transparently as a guarantee of diagnostic quality, not as a refusal or a delay.

### 9.2 Longitudinal re-evaluation and authentic comorbidity

The ASD diagnosis, like any complex psychiatric diagnosis, should not be considered permanent and irrevocable. Institutional willingness to re-examine it in light of treatment response and clinical evolution is an essential component of responsible diagnostic practice. Authentic comorbidity between ASD and Personality Disorders is documented in the literature (53): the emotional regulation difficulties inherent to ASD may favour, in the presence of cumulative trauma or dysfunctional primary relationships, the development of dysfunctional personality organisations. In these cases, the assessment must identify which component is primary and which secondary, orienting treatment priority accordingly.

## 10 Toward minimum evidentiary standards in adult ASD assessment

The argument developed in Sections 1 to 9 converges on a single core question: what minimum evidentiary standards should govern adult ASD differential diagnosis in complex cases? Section 10 sets out the answer in two complementary forms — an ideal six-component protocol (Sections 10.1–10.7) and a minimum feasible standard intended for resource-constrained settings (Section 10.8), summarised together in **Table 1**.

### 10.1 The crisis of the cross-sectional single-source model

The diagnostic problem discussed in preceding sections is not only categorical — which label to apply — but profoundly methodological: how the diagnosis is reached, with which instruments, over which time frame, and through how many sources of information. Current clinical practice in adult ASD assessment is often characterised by cross-sectional evaluation conducted in one or two sessions, based predominantly on self-report and self-administered questionnaires, with developmental history collected non-systematically. This model is structurally inadequate to the complexity of the diagnostic problem that ASD poses, particularly in cases where differential diagnosis with Personality Disorders is not clinically evident and most acutely in the Type II population identified in Section 5b.

Frith (13) noted that sample contamination depends substantially on the imprecision of the evaluative process: diagnoses based on self-report questionnaires with high sensitivity and low specificity, in the absence of direct behavioural observation and systematic developmental history collection, produce heterogeneous diagnostic populations that make it difficult to identify the neurocognitive mechanisms specific to autism. The same principle applies with equal force to clinical practice: a methodologically fragile diagnosis is a high-error-risk diagnosis, with therapeutic consequences measurable in years or decades of a person’s life.

### 10.2 Temporal dimension: longitudinal stability as diagnostic criterion

DSM-5 explicitly requires that ASD symptoms be “present in the early developmental period” — a criterion that in adult evaluation is too often reduced to a generic question about childhood, collected from the subject without verification by independent informants. Longitudinal trait stability is in fact one of the most powerful diagnostic elements available to the clinician: genuine autistic traits — social reciprocity difficulty, restricted interests, pragmatic peculiarities, multimodal sensory profile — display continuity across time and contexts that does not characterise most Personality Disorders, whose manifestations are typically modulated by relationship quality and stress intensity.

This implies that ASD diagnosis in adulthood requires a developmental history conducted with methodological rigour comparable to a structured interview: systematic collection of information across multiple domains (language, social reciprocity, interests, sensoriality, executive function), across multiple developmental phases (early schooling, developmental age, adolescence), through multiple independent informants (parents, teachers, siblings, partners), and where possible through documentary materials (photographs, family videos, school reports, previous assessments). The Autism Diagnostic Interview—Revised (ADI-R; (54)) remains the international gold-standard instrument for this function, but its systematic use in routine clinical practice remains largely insufficient.

### 10.3 Direct behavioural observation: limits of gold-standard instruments

The ADOS-2 (55) is considered the gold-standard instrument for ASD behavioural observation and has been validated on large clinical samples. However, it presents methodological limitations when applied to the diagnostically most complex profiles — particularly cognitively high-functioning adults with possible female phenotype, who correspond largely to the Type II population. The ADOS-2 standardised situation elicits social behaviours in an interaction with an unfamiliar examiner: this context produces ASD-typical difficulties in affected subjects, but is also a context in which camouflaging can be effectively activated, rendering observation less sensitive in individuals with high conscious adaptation capacities. Lord and colleagues (3) and Volkmar and McPartland (14) acknowledged that the ADOS-2’s inter-rater reliability in real clinical contexts is substantially lower than that obtained in validation trials with specialised evaluators.

The response to this limitation is not the abandonment of the ADOS-2, but its use as one component of a more articulated evaluative process that includes naturalistic observation in multiple contexts, qualitative analysis of communicative pragmatics in unstructured situations, and behavioural micro-analysis of interactional sequences. The clinical-formulation model — integrating standardised instrument scores with longitudinal clinical reasoning and understanding of the person’s overall functioning — should supersede the threshold-scoring diagnostic model, which reduces assessment to a scoring operation without qualitative depth.

### 10.4 Granularity of sensory assessment: beyond the checklist

The sensory profile is assessed in clinical practice predominantly through self-administered questionnaires — Short Sensory Profile (SSP), Sensory Processing Measure (SPM), Sensory Over-Responsivity Inventory (SensOR) — that measure the frequency and intensity of subjectively reported symptoms without distinguishing between different underlying mechanisms. This approach produces information useful at a screening level, but insufficient for differential diagnosis. The intra-categorical heterogeneity documented by Kadlaskar and colleagues (35) — with four distinct sensory classes within the same ASD population — demonstrates that an aggregate measure of sensory profile obscures clinically and biologically significant differences requiring instruments of superior granularity.

A methodologically refined sensory assessment should distinguish at least the following dimensions: (a) sensory modality involved, since in ASD the profile is typically multimodal whilst in anxious or traumatic conditions it is more selective; (b) direction of reactivity (hyper-responsivity vs. hypo-responsivity vs. sensation seeking), since the different directions have distinct neurophysiological correlates and clinical implications; (c) contextual stability — whether sensory reactivity is present stably in emotionally neutral contexts or appears exclusively in states of elevated affective activation; (d) presence since early childhood, reconstructable through history with multiple informants. Psychophysiological measures — event-related potentials (ERPs), skin conductance, and eye-tracking — are emerging as instruments capable of objectifying aspects of the sensory profile not accessible to subjective self-report (32), although their clinical use remains largely experimental.

### 10.5 The Marrian three-level model in psychiatric nosology

The theoretical model originally articulated by Marr (56) and applied to developmental psychopathology by Morton and Frith (57) analyses any complex disorder across three distinct levels: the biological (genetics, neurobiology, cerebral physiology), the cognitive (information-processing mechanisms, theory of mind, central coherence, executive function), and the behavioural (observable symptoms, daily functioning, relationships). A methodologically adequate diagnosis should aspire to describe the person in relation to all three levels, recognising that a purely behavioural diagnosis — based exclusively on symptom observation — is necessarily underspecified. This three-level framework is widely used in cognitive psychopathology and is not specific to ASD; it is invoked here because the empirical case for internal heterogeneity in ASD (Section 5b) is most compelling when articulated across all three levels.

In current clinical practice, the biological level is almost always absent — in the absence of validated biomarkers — and the cognitive level is assessed partially and non-systematically. Diagnostic granularity should increase convergence across available levels: neuropsychological assessment of the cognitive profile (executive function, central coherence, mentalising, working memory), qualitative analysis of communicative pragmatics, evaluation of information processing strategies in structured tasks. Convergence of indicators from different analytical levels — a cognitive profile consistent with impaired theory of mind and weak central coherence, combined with a stable multimodal sensory profile and documented developmental history — produces a substantially more defensible diagnosis than one based exclusively on the threshold score of a self-administered questionnaire.

### 10.6 Informant discordance as diagnostic data

A systematically undervalued element in clinical practice is discordance between scores produced by different informants: subject self-report, parental report, clinician observation, and teacher assessment (58). In ASD research, discordance between self-report and hetero-report has been extensively documented: subjects with ASD tend to under-report their difficulties in some domains (emotional reciprocity, executive function) and over-report in others (sensoriality, internal state), likely related to the introspection difficulties and alexithymia frequently associated with the condition. In subjects with BPD, the inverse pattern is observed: self-report amplifies the intensity of subjective suffering whilst external observation reveals functioning often superior to that described.

Systematising informant discordance — not as a diagnostic obstacle to be resolved but as clinically informative data to be analysed — can constitute a differentiating instrument of considerable value. A systematic pattern of elevated self-report of atypical sensoriality in the absence of confirmation from independent informants and documented developmental history should raise the diagnostic threshold for ASD, not lower it. Conversely, a pattern of pragmatic and sensory difficulties consistently documented by multiple informants across development — even in the absence of a prior formal diagnosis — has high confirmatory value.

### 10.7 An integrated protocol: six minimum quality standards (ideal)

Based on the methodological considerations developed above, ASD diagnostic evaluation in adulthood — particularly in cases of complex differential diagnosis — should meet six minimum quality standards: structured developmental history with at least two independent informants; multi-context behavioural observation; neuropsychological profiling where indicated; granular sensory assessment by modality, direction, and contextual stability; systematic evaluation of alternative diagnoses; and longitudinal clinical formulation with revisability. This model is not a guarantee of infallibility: ASD diagnosis will remain probabilistic in the absence of validated biological biomarkers, and hypothesis revision over time will remain an indispensable component of responsible practice. It is, however, a system that systematically reduces error risk, increases convergence amongst evidence sources, and produces clinically more stable and therapeutically more useful diagnoses. **Table 1** presents the ideal and minimum feasible standards in operational form.

### 10.8 Minimum feasible versus ideal standards: notes on international applicability

The six minimum evidentiary standards described in Section 10.7 are formulated as an ideal: they presuppose access to multiple independent informants, time for multi-context observation, specialised instrumentation (ADOS-2, ADI-R), neuropsychological infrastructure, and integrated care pathways across psychiatric subspecialties. These resources are not uniformly available. Fragmented or under-resourced systems — including but not limited to many non-Western and Global South contexts, but also rural and community mental health services in high-income countries — face workforce shortages, limited access to ADOS/ADI-based assessment, and weak integration between psychiatry and developmental services. Recommendations developed without acknowledgement of this asymmetry risk becoming aspirational rather than operational.

We therefore articulate a minimum feasible standard alongside the ideal one. The minimum feasible standard is intended to be implementable in resource-constrained services without specialised ASD instrumentation, and consists of four components: (i) developmental history collected from at least one independent informant, with explicit documentation of the absence or inaccessibility of further informants where this is the case; (ii) systematic consideration of at least three alternative diagnoses appropriate to the clinical presentation (typically including at least one Personality Disorder, one trauma-related diagnosis, and one mood or attentional condition), with documentation of why each is retained or excluded; (iii) a documented decision not to close the diagnosis at the first session if any of the three alternatives remains unresolved; (iv) a follow-up appointment scheduled within a defined time window for re-evaluation in light of clinical evolution and any additional information that has become available.

The minimum feasible standard is not a concession to under-resourced systems but a clinically defensible response to them. It preserves the substantive principle of the ideal protocol — that an ASD diagnosis should not be closed in the presence of unresolved alternatives — whilst recognising that the operational form of that principle depends on resources actually available. Where alternative services exist, parallel referral remains the preferred route; where they do not, provisional formulation and continued follow-up within the assessing service remain available. Denial of care is never an appropriate response to diagnostic uncertainty.

Two further considerations matter for international applicability. First, the cultural calibration of behavioural and pragmatic norms differs across populations, and assessment instruments validated in Western European and North American samples may produce systematic biases in other contexts. Second, family structure and informant availability vary across societies, and the requirement of independent informants must be adapted to local kinship patterns without abandoning the substantive principle of multi-source evidence. The minimum feasible standard is intended to be locally adaptable in these respects, and we do not propose a one-size-fits-all operationalisation. The principle is methodological convergence across as many sources as a given system can provide, not a fixed number of instruments.

## 11 Limitations

This review has several limitations that must be acknowledged. First, as a narrative review, it is susceptible to selection bias in the literature reviewed and does not follow the systematic procedures that would reduce such bias; the methodological note in Section 1.3 is intended to make the literature-identification process auditable but does not substitute for systematic-review procedures. Second, the clinical indicators proposed for differential assessment (Section 8.9) and the integrated protocol (Sections 10.7–10.8) are expert-derived recommendations based on the reviewed literature and clinical observation, not empirically validated guidelines. Their operationalisation and validation in prospective clinical studies is a necessary next step. Third, the focus on adult assessment and on the female phenotype, whilst clinically significant, does not exhaust the range of conditions and populations posing diagnostic challenges to ASD assessment; paediatric assessment, intellectual-disability comorbidity, and culturally heterogeneous samples deserve dedicated treatment. Fourth, the Type I/Type II partition introduced in Section 5b is heuristic rather than formally codified, and its operational boundaries remain to be settled empirically. Fifth, the BPD/ASD sensory contrast described in Sections 6.3 and 8.2 is a working clinical heuristic supported by convergent observational data, not yet validated by head-to-head psychophysical studies.

### 11.1 Counter-arguments and their engagement

Three counter-arguments to the position developed here deserve explicit engagement, in line with a balanced approach to the debate.

#### 11.1.1 The improved-case-finding objection

It can be argued that the prevalence increase reflects genuine improved case-finding in previously underserved populations — women, adults, ethnic and linguistic minorities — rather than diagnostic drift. This is partly correct and the data discussed in Section 3 explicitly acknowledge it. The argument of the present review is not that improved case-finding has not occurred, but that it cannot account for the magnitude of the increase observed in the United States and Northern Europe (1, 23, 24), and that the ASD-Type II population identified through better case-finding overlaps phenomenologically with several non-ASD conditions in ways that produce systematic error. Improved sensitivity has been accompanied, not offset, by reduced specificity. The dual-risk framing introduced in Section 1.2 is the explicit recognition that both errors matter.

#### 11.1.2 The neurodiversity objection

It can be argued that any tightening of diagnostic boundaries risks undoing the civil and clinical gains produced by the neurodiversity framework: reduced stigma, positive identity narratives, reasonable accommodation, and — most importantly — the recognition of populations historically missed. The position taken here is that diagnostic accuracy is a precondition for, rather than an obstacle to, those gains. A diagnostic label applied with low specificity loses its capacity to identify a population for which evidence-based interventions exist; it also loses public credibility, which ultimately damages the individuals who carry the diagnosis legitimately. Restoring specificity protects the neurodiversity framework rather than undoing it, provided the operational forms of tightening (stepped triage, provisional formulation, minimum feasible standards) do not become institutional gatekeeping (Section 9.1).

#### 11.1.3 The resource-and-access objection

It can be argued that the methodological recommendations developed in Section 10 are resource-intensive and may further restrict access to assessment in already under-resourced services. This is a legitimate concern that we share, and it has shaped the structure of Section 10.8. The recommendations are framed as minimum quality standards for complex differential-diagnostic cases, not as requirements for all assessments. Triage protocols can identify cases in which simpler assessment is sufficient (clear early-onset Type I presentations with classical phenomenology) and reserve the integrated protocol for genuinely uncertain cases. For systems in which even the ideal protocol is structurally inaccessible, the minimum feasible standard articulated in Section 10.8 offers an implementable alternative that preserves the substantive principle.

## 12 Conclusion

Current ASD assessment, particularly in complex adult presentations, faces a dual challenge: improving recognition of historically under-identified individuals whilst protecting diagnostic validity in cases with substantial overlap with personality, trauma-related, and anxiety-related conditions. This dual challenge cannot be met by tightening or loosening the criteria in isolation, nor by amplifying or restraining the neurodiversity framework in isolation. It requires the substantive principle that an ASD diagnosis should be applied when developmental, behavioural, and differential evidence converge sufficiently — and that the operational form of that principle should be calibrated to the resources actually available in the service in which the assessment occurs.

The recent neurobiological evidence on internal heterogeneity, summarised in Section 5b as the Type I/Type II partition and supported by the Litman et al. (6) person-centred analysis, anchors the clinical argument: the diagnostic category is internally heterogeneous in ways that are unlikely to be clinically inert, and the population most affected by the methodological limitations of routine assessment is precisely the Type II population in which differential diagnosis with general psychopathology is most challenging.

The sensory profile, formally included in DSM-5 since 2013, is clinically relevant but diagnostically non-specific: it occurs with high frequency in Anxiety Disorders, Complex PTSD, BPD, and the normative Highly Sensitive Person variant. Its intra-categorical heterogeneity in ASD — with 41% of diagnosed individuals displaying sensoriality broadly within normal limits (35) — further reduces its discriminative value. The most clinically useful differentiating element, with the caveats noted in Section 6.3, is selectivity: pervasive and multimodal in ASD, more selectively interpersonal and contingent on emotional state in BPD. This contrast is accessible only through a sensory assessment of adequate granularity — not through a self-administered questionnaire in a single session.

The integrated assessment protocol proposed in Section 10.7, together with the minimum feasible standard articulated in Section 10.8, represents a two-tier system of methodological quality for complex differential-diagnostic cases. Its adoption — in whichever of the two forms is locally feasible — would reduce systematic diagnostic-error risk, increase evidence-source convergence, and restore to the ASD diagnosis the therapeutic relevance that diagnostic drift has progressively eroded.

The epistemological model that the field as a whole has been moving towards — willingness to revise positions in light of data, including positions one has personally advocated — is the model that clinical practice should adopt. Not irrevocable diagnostic certainties, but hypotheses submitted to continuous verification, through instruments of increasing granularity and a clinical observation that treats the diagnosis as a working model rather than a permanent verdict.
